# Oxidative Stress to Promote Cell Death or Survival

**DOI:** 10.1155/2016/2054650

**Published:** 2016-01-31

**Authors:** Michela Battistelli, Manuela Malatesta, Stefania Meschini

**Affiliations:** ^1^Department of Earth, Life and Environmental Sciences, University of Urbino Carlo Bo, Campus Scientifico “E. Mattei”, Via Ca' le Suore 2, 61029 Urbino, Italy; ^2^Department of Neurological and Movement Sciences, Anatomy and Histology Section, University of Verona, Strada le Grazie 8, 37134 Verona, Italy; ^3^Department of Technology and Health, Istituto Superiore di Sanità, Viale Regina Elena 299, 00161 Rome, Italy

Oxidative stress is one of the mechanisms through which cells respond by activating pathways of cell survival or programmed cell death. The initial response of the cell to a stressful stimulus is activated for helping the cell to defend itself and respond positively to the insult. If the insult is very harmful and unresolved, it is the activation of programmed cell death by the cell itself with the aim of eliminating damaged cells without the presence of the inflammatory process.

The survival of a cell depends on the ability to activate an appropriate response to environmental and intracellular stimuli; this explains why the reaction is very conserved in evolution. In fact, the defense mechanisms of the cell through the activation of antioxidant systems against oxidative damage or stress proteins as the “heat shock proteins” occur both in lower organisms and in mammals.

The cells can be subjected to different types of stress and the response of the cells depends on the type and the level of insult.

For example, the attempt of survival is induced by a cell in the case of the heat shock response or the unfolded proteins response leading to activation of chaperone protein that increases the ability of the protein to fold, by counteracting induced stress and so promoting the cell survival. Thus, it is the adaptability of a cell to define its fate.

The cell is able to activate various cellular defense mechanisms and survival according to the level and mode of stress. If, however, these mechanisms are not activated successfully, then different types of cell death are promoted. The main mechanisms of cell death are apoptosis, necrosis, pyroptosis, and autophagic cell death. The activation of one mechanism of death or another depends on the cellular capacity to deal with the condition of suffering to which it is exposed. In this section, we wanted to learn about these topics:

(i) T. Yan et al. studied the effect of excessive alcohol consumption on brain tissue damage and cognitive dysfunction. They have shown that heavy drinking is associated with an earlier onset of neurodegenerative diseases such as Alzheimer's disease. They concluded that acetaldehyde induces cytotoxicity of SH-SY5Y cells via promotion of apoptotic signaling, inhibition of cell survival pathway, and induction of oxidative stress. So the inhibition of oxidative stress by antioxidants may be beneficial for preventing neuronal damage associated with acetaldehyde-induced cytotoxicity which could result from excessive alcohol consumption.

(ii) L. S. de Castro et al. analyzed the influence of oxidative status on spermatozoa by distinct mechanisms, from capacitation to fertilization.

They concluded that sperm, when exposed to oxidative environment, may present impaired motility traits, prooxidative status, and premature capacitation; such alterations resulting from embryo development fail, from the first cleavage to blastocyst.

(iii) T. Dou et al. assumed that herbicides and pesticides have been linked to nigrostriatal damage and the emergence of Parkinson symptoms in epidemiological and animal studies and have investigated the oxidative stress effect of paraquat (PQ), a widely used herbicide in the world, on immortalized human embryonic neural progenitor cells (hNPCs).

They demonstrated that PQ exposure could significantly induce oxidative stress and cause oxidative imbalance.

(iv) S. Bekeschus et al. have studied the plasma composition and the beneficial role of cold plasmas in human pathologies such as wound healing. These studies demonstrated an effective antioxidant power by the cold blood cells subjected to inflammatory process.

(v) L. Dyugovskaya et al. wanted to elucidate the conditions and mechanisms involved in G*φ* formation. G*φ* formation may provide insights into basic neutrophil biology in inflammatory and atherogenic conditions or in the resolution of neutrophilic inflammation. Moreover, the a priori low yield of G*φ* may indicate that they have a unique function and may represent a subgroup of progenitor cells.

(vi) X. Tang et al. assumed that the human Circadian Locomotor Output Cycle protein Kaput (CLOCK) gene was originally discovered as a regulator of essential human daily rhythms. They investigated the role of hCLOCK in the hypoxia-oxidative stress response system at the biochemical level.

They demonstrated that hypoxic states induced vascular oxidative damage and inflammation via hCLOCK-mediated production of ROS, with subsequent activation of the RhoA and NF-*κ*B pathways.

This special issue treated, in a multidisciplinary way, the oxidative stress topic.

*Michela Battistelli* *Michela Battistelli* *Manuela Malatesta* *Manuela Malatesta* *Stefania Meschini* *Stefania Meschini*

